# Novel Biologically Potent Diorganosilicon(IV) Complexes of Indole-2,3-Dione Derivatives

**DOI:** 10.1155/BCA.2005.255

**Published:** 2005

**Authors:** R. V. Singh, Pooja Nagpal

**Affiliations:** Department of Chemistry University of Rajasthan Jaipur 302 004 India

## Abstract

The aim of the present study is to synthesize some novel ecofriendly fungicides and bactericides of indole-2,3-dione derivatives, having important pharmacodynamic significance. The ligands used in the present account are derived by the condensation of 1,3-dihydro-3-[2-(phenyl)-2-oxoethylidene]-2H-indol-2- one, 1,3-dihydro-3-[2-(4-nitrophenyl)-2-oxoethylidene]-2H-indol-2-one and 1,3-dihydro-3-[2-(4-nitro-3-methylphenyl)- 2-oxoethylidene]-2H-indol-2-one with hydrazinecarboxamide and hydrazinecarbothioamide. These imines, on interaction with diorganosilicon(IV) chlorides, yield complexes having Si–O or Si–S and Si←N bonds. The structure of these compounds have been elucidated by elemental microanalyses and spectral [(UV), (IR), ^1^H, ^13^C and ^29^Si NMR)] studies which unerringly point to a trigonal bipyramidal and octahedral geometries for unimolar and bimolar reactions, respectively. The potency of the synthesized compounds have been assessed by growth inhibiting potential of the complexes against variety of fungal and bacterial strains and male albino rats. The results of these biological studies have been compared with the standard fungicide, Bavistin. The studies demonstrate that, 1,3-dihydro-3-[2-(4-nitrophenyl)-2-oxoethylidene]-2H-indol-2-onehydrazincarbothioamide and its diphenylsilicon(IV) complexes have comparable antimicrobial activity and are less toxic to male albino rats than Bavistin.


### Novel Biologically Potent Diorganosilicon(IV) Complexes of Indole-2,3-Dione Derivatives

R.V. Singh* and Pooja Nagpal

*Department of Chemistry, University of Rajasthan,*
*Jaipur-302 004, India*

### ABSTRACT

The aim of the present study is to synthesize some novel ecofriendly fungicides and bactericides of indole-2,3-dione derivatives, having important pharmacodynamic significance. The ligands used in the present account are derived by the condensation of 1,3-dihydro-3-[2-(phenyl)-2-oxoethylidene]-2H-indol-2-one, 1,3-dihydro-3-[2-(4-nitrophenyl)-2-oxoethylidene]-2H-indol-2-one and 1,3-dihydro-3-[2-(4-nitro-3-methyl-phenyl)-2-oxoethylidene]-2H-indol-2-one with hydrazinecarboxamide and hydrazinecarbothioamide. These imines, on interaction with diorganosilicon(IV) chlorides, yield complexes having Si–O or Si–S and Si←N bonds. The structure of these compounds have been elucidated by elemental microanalyses and spectral [(UV), (IR), $^{1}H$, $^{13}C$ and $^{29}Si$ NMR)] studies which unerringly point to a trigonal bipyramidal and octahedral geometries for unimolar and bimolar reactions, respectively. The potency of the synthesized compounds have been assessed by growth inhibiting potential of the complexes against variety of fungal and bacterial strains and male albino rats. The results of these biological studies have been compared with the standard fungicide, Bavistin. The studies demonstrate that, 1,3-dihydro-3-[2-(4-nitrophenyl)-2-oxoethylidene]-2H-indol-2-one-hydrazincarbothioamide and its diphenylsilicon(IV) complexes have comparable antimicrobial activity and are less toxic to male albino rats than Bavistin.

### INTRODUCTION

The biochemistry of synthetic organometallics has generated active research related to their biochemical significances. Extensive literature on the biological properties of many semicarbazones /1,2/ and thiosemicarbazones /3,4/ are available and new examples continue to be tested for their antitumour and anti-HIV activity /5,6/. Reports have appeared on the antiviral activity of several isatin-3-thiosemicarbazones /7,8/ which has stimulated isatin to be screened for a wide range of biological effects both in animals /9/ and in plants /10/. *In vivo* studies have indicated that some biologically active compounds may become more carcinostatic and bacteriostatic upon chelation /11-14/. The interest in organosilicon(IV) compounds is

* Author for correspondence : E-mail : kudiwal@datainfosys.net generated due to their versatile applicability in pharmaceutical and in chemical industries. For example, use of very bulky silicon-containing ligands allows the isolation of a wide range of previously inaccessible types of compounds and silicon substituted methyl groups are capable of making considerable adjustments, especially in the inner $CSi_3$ skeleton in response to the electronic demands of the adjacent elements /15/.

Encouraged by the above findings and our interest in the biological and chemical properties of such compounds, synthesis and spectroscopic characterization of several new silicon complexes of monofunctional bidentate ligands derived from semicarbazones and thiosemicarbazones of indole-2,3-dione derivatives have been studied. The imines used during these studies are shown in Fig. 1. An extensive evaluation of the toxicology of these compounds against variety of fungal and bacterial strains and also on male albino rats has also been conducted.

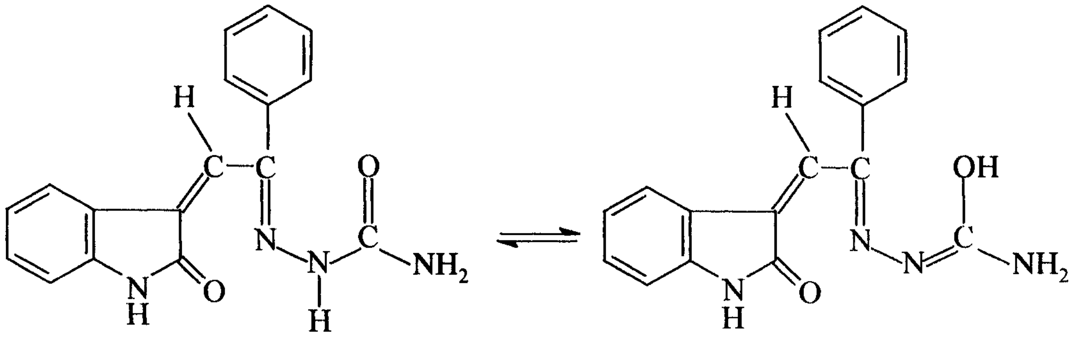

1,3-Dihydro-3-[2-(phenyl)-2-oxoethylidene]-2H-indol-2-one-hydrazine carboxamide ($L_1H$)

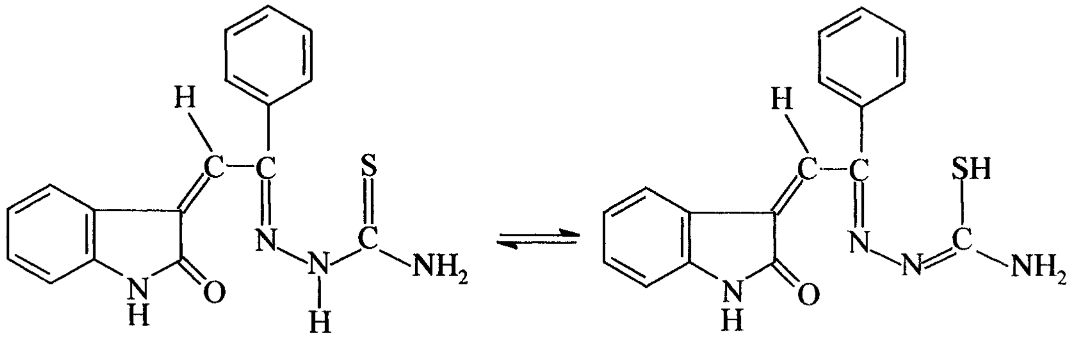

1,3-Dihydro-3[2-(phenyl)-2-oxoethylidene]-2H-indol-2-one-hydrazinecarbothioamide ($L_2H$)

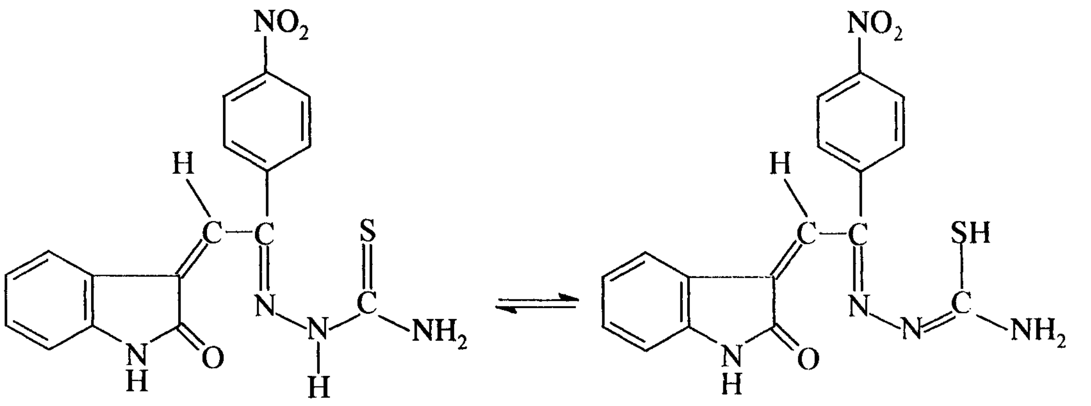

1,3-Dihydro-3-[2-(4-nitrophenyl)-2-oxoethylidene]-2H-indol-2-one-hydrazine-carbothioamide ($L_3H$)

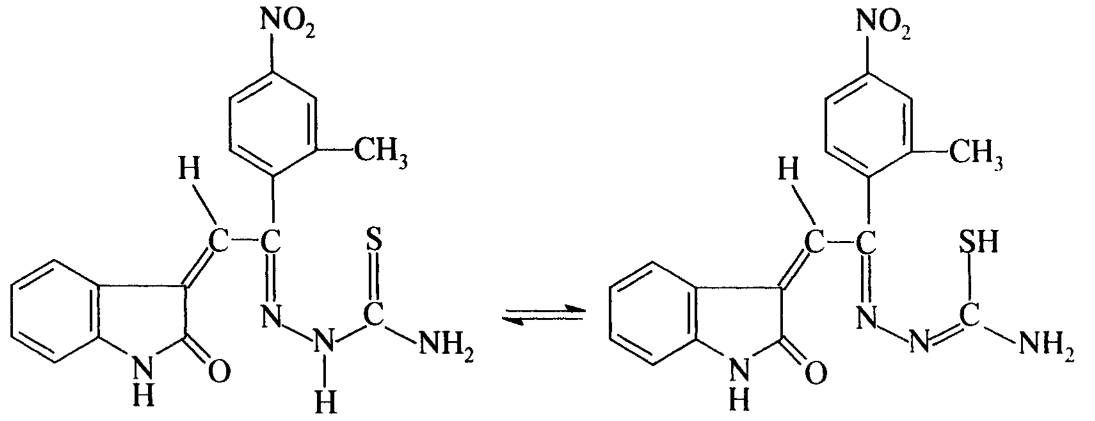

1,3-Dihydro-3-[2-(4-nitro-3-methylphenyl)-2-oxoethylidene]-2H-indol-2-one-hydrazinecarothioamide ($L_4H$)

**Fig. 1**

### RESULTS AND DISCUSSION

The 1 : 1 and 1 : 2 molar reactions of $Ph_2SiCl_2$ and $Me_2SiCl_2$ with semicarbazone and thiosemicarbazone ligands have led to the formation of $Ph_2SiCl(\widehat{N\ X})$, $Ph_2Si(\widehat{N\ X})_2$ $Me_2SiCl(\widehat{N\ X})$ and $Me_2Si(\widehat{N\ X})_2$ types of complexes. The reactions have been carried out in dry methanol and proceed smoothly with the precipitation of NaCl. These reactions can be represented by the following general equations:

$$R_2SiCl_2 + \widehat{N\ X}.Na \xrightarrow{1:1} R_2SiCl(\widehat{N\ X}) + NaCl$$

$$R_2SiCl_2 + 2\widehat{N\ X}.Na \xrightarrow{1:2} R_2Si(\widehat{N\ X})_2 + 2NaCl$$

(where, $\widehat{N\ X}$ is the donor system of the ligands and X = O or S; R = Ph or Me).

The newly synthesized derivatives are coloured solids, with sharp melting points (Table I), soluble in common organic solvents and susceptible to moisture. The bonding pattern of these monomeric non-electrolytes (10–15 $\Omega^{-1}$ $cm^2$ $mol^{-1}$ in dry dimethylformamide) has been deduced on the basis of infra red and multinuclear NMR ($^1H$, $^{13}C$ and $^{29}Si$) spectroscopic studies.

### IR Spectra

In the IR spectra (Table II) of the ligands, a broad band in the region 3250-3100 $cm^{-1}$ may be assigned to ν(NH) vibrations. However, in the solution spectra, an additional band due to ν(SH) also appear at ~ 2525 $cm^{-1}$ due to tautomerization /16/. The ν(NH) or ν(SH) bands disappear in the spectra of the resulting complexes indicating the possible deprotonation of the ligands on complexation and the formation of the (Si–S) and (Si←N) bonds. A sharp band at 1613 $\pm$ 2 $cm^{-1}$ due to (>C=N) frequency of the free azomethine group in the ligands shifts to the lower frequency (~ 20 $cm^{-1}$) in the silicon complexes indicating the coordination of the azomethine nitrogen to the silicon atom. In the dimethylsilicon(IV) derivatives, a band at ~ 1420 $cm^{-1}$ has been ascribed to the asymmetric deformation vibrations of ($CH_3$–Si) /17/ group whereas the band at ~1270 $cm^{-1}$ is assigned to the symmetric mode of ($CH_3$–Si) group. Several new bands are observed in the spectra of the complexes at ~ 620 $cm^{-1}$/540 $cm^{-1}$ and 580 $cm^{-1}$ and these are due to ν(Si–O)/ν(Si–S) /18/ and ν(Si←N) /19/ vibrations, respectively. A band due to ν(Si–Cl) /20/ at 512 $cm^{-1}$ is observed in 1:1 diorganosilicon(IV) derivatives. There are, however, no changes in the $\nu_{sym}$ and $\nu_{asym}$ modes of $NO_2$ group appearing at ca. 1345 $cm^{-1}$ and 1520 $cm^{-1}$, respectively /21/, in the ligands. The presence of one (Si←N) band in the 1:2 complexes suggests that the complexes exist in the trans form.

### $^1$H NMR Spectra

The mode of bonding discussed above is further supported by comparing the $^1H$ NMR spectra of the ligands with the diorganosilicon(IV) complexes (Table III).The spectra of the ligands display broad signals due to the NH protons which disappear in the silicon complexes indicating the coordination of the azomethine nitrogen as well as covalent bond formation between silicon and oxygen/sulphur due to the deprotonation of the enolic form of the ligand. Further, in the spectra of the complexes, a downfield shift in the position of the aromatic protons again indicates the proposed coordination. The appearance of the signal due to the $NH_2$ group at about the same position in the ligands and their silicon complexes shows the non-involvement of this group in coordination. Further, the additional signals in the region δ 0.72–0.98 ppm are due to $Me_2Si$ groups.

### $^{13}$C NMR Spectra

The $^{13}C$ NMR spectra of the ligands and their corresponding silicon complexes have been recorded in dry methanol (Table IV). The chemical shift values of the carbon atoms attached with the azomethine nitrogen

**Table 1 :**

Physical Properties and Analytical Data of the Organosilicon(IV) Complexes of ( $\widehat{O\ N}$ ) and ( $\widehat{S\ N}$ ) Donor Ligands Derived from Indol-2,3-dione Derivatives

| Reactant g (mmol) | | | Molar | Molecular | M.P. | Elemental analyses (%)[a] | | | | | | Mol. |
|---|---|---|---|---|---|---|---|---|---|---|---|---|
| M | Ligand | Na | ratio | Formulae (Colour and state) | (°C) | C | H | N | S | Cl | Si | Wt.[a] |
| $Ph_2SiCl_2$ | $L_1H$ | | | | | | | | | | | |
| 0.72 | 0.87 | 0.065 | 1:1 | $C_{29}H_{23}N_4O_2SiCl$ | 94 | 66.45 | 4.35 | 10.56 | – | 6.65 | 5.28 | 506 |
| (2.84) | (2.84) | (2.84) | | Reddish brown solid | | (66.59) | (4.43) | (10.71) | | (6.78) | (5.37) | (523.06) |
| $Ph_2SiCl_2$ | $L_1H$ | | | | | | | | | | | |
| 1.07 | 2.59 | 0.194 | 1:2 | $C_{46}H_{36}N_8O_4Si$ | 206 | 69.54 | 4.43 | 14.02 | – | - | 3.41 | 776 |
| (4.22) | (8.46) | (8.45) | | Red solid | | (69.68) | (4.58) | (14.13) | | | (3.54) | (792.92) |
| $Me_2SiCl_2$ | $L_1H$ | | | | | | | | | | | |
| 0.84 | 1.99 | 0.149 | 1:1 | $C_{19}H_{19}N_4O_2SiCl$ | 87 | 57.06 | 4.69 | 13.88 | – | 8.82 | 6.89 | 378 |
| (6.51) | (6.51) | (6.49) | | Red solid | | (57.21) | (4.80) | (14.04) | | (8.89) | (7.04) | (398.94) |
| $Me_2SiCl_2$ | $L_1H$ | | | | | | | | | | | |
| 0.65 | 3.08 | 0.231 | 1:2 | $C_{36}H_{32}N_8O_4Si$ | 165 | 64.15 | 4.66 | 16.78 | – | – | 4.05 | 654 |
| (5.04) | (10.07) | (10.05) | | Brick red solid | | (64.27) | (4.79) | (16.65) | | | (4.17) | (668.78) |
| $Ph_2SiCl_2$ | $L_2H$ | | | | | | | | | | | |
| 0.75 | 0.95 | 0.067 | 1:1 | $C_{29}H_{23}N_4OSSiCl$ | 176 | 64.46 | 4.18 | 10.22 | 5.86 | 6.49 | 5.09 | 522 |
| (2.96) | (2.96) | (2.93) | | Crystalline red solid | | (64.61) | (4.30) | (10.39) | (5.95) | (6.58) | (5.21) | (539.13) |
| $Ph_2SiCl_2$ | $L_2H$ | | | | | | | | | | | |
| 0.96 | 2.44 | 0.173 | 1:2 | $C_{46}H_{36}N_8O_2S_2Si$ | 180 | 66.85 | 4.31 | 13.46 | 7.65 | – | 3.29 | 807 |
| (3.79) | (7.58) | (7.54) | | Brown solid | | (66.97) | (4.40) | (13.58) | (7.77) | | (3.40) | (825.06) |
| $Me_2SiCl_2$ | $L_2H$ | | | | | | | | | | | |
| 0.88 | 2.20 | 0.156 | 1:1 | $C_{19}H_{19}N_4OSSiCl$ | 162 | 55.84 | 4.55 | 13.62 | 7.58 | 8.48 | 6.63 | 398 |
| (6.82) | (6.82) | (6.79) | | Red solid | | (55.99) | (4.61) | (13.50) | (7.73) | (8.54) | (6.77) | (414.99) |
| $Me_2SiCl_2$ | $L_2H$ | | | | | | | | | | | |
| 0.67 | 3.35 | 0.238 | 1:2 | $C_{36}H_{32}N_8O_2S_2Si$ | 160 | 6.08 | 4.49 | 15.83 | 9.03 | – | 3.89 | 683 |
| (5.19) | (10.38) | (10.35) | | Brick red solid | | (6.17) | (4.60) | (15.99) | (9.15) | | (4.01) | (700.92) |
| $Ph_2SiCl_2$ | $L_3H$ | | | | | | | | | | | |
| 0.78 | 1.13 | 0.071 | 1:1 | $C_{29}H_{22}N_5O_3SSiCl$ | 115 | 59.55 | 3.88 | 11.83 | 5.36 | 5.96 | 4.69 | 569 |
| (3.09) | (3.08) | (3.10) | | Crystalline orange solid | | (59.63) | (3.79) | (11.99) | (5.49) | (6.07) | (4.81) | (584.13) |

| Reactant g (mmol) | | | Molar | Molecular | M.P. | Elemental analyses (%)[a] | | | | | | Mol. |
|---|---|---|---|---|---|---|---|---|---|---|---|---|
| M | Ligand | Na | ratio | Formulae (Colour and state) | (°C) | C | H | N | S | Cl | Si | Wt.[a] |
| $Ph_2SiCl_2$ | $L_3H$ | | | | | | | | | | | |
| 0.78 | 2.26 | 0.142 | 1:2 | $C_{46}H_{34}N_{10}O_6S_2Si$ | 290 | 60.24 | 3.62 | 15.50 | 6.87 | – | 3.18 | 901 |
| (3.08) | (6.16) | (6.19) | | Light orange solid | | (60.38) | (3.74) | (15.31) | (7.01) | | (3.07) | (915.06) |
| $Me_2SiCl_2$ | $L_3H$ | | | | | | | | | | | |
| 0.82 | 2.33 | 0.147 | 1:1 | $C_{19}H_{18}N_5O_3SSiCl$ | 112 | 49.52 | 3.81 | 15.10 | 6.82 | 7.64 | 6.85 | 436 |
| (6.35) | (6.35) | (6.39) | | Orange solid | | (49.61) | (3.94) | (15.23) | (6.97) | (7.71) | (6.97) | (459.99) |
| $Me_2SiCl_2$ | $L_3H$ | | | | | | | | | | | |
| 0.61 | 3.47 | 0.219 | 1:2 | $C_{36}H_{30}N_{10}O_6S_2Si$ | 178 | 54.55 | 3.71 | 17.66 | 8.30 | – | 3.43 | 775 |
| (4.73) | (9.45) | (9.51) | | Orange solid | | (54.67) | (3.82) | (17.71) | (8.11) | | (3.55) | (790.92) |
| $Ph_2SiCl_2$ | $L_4H$ | | | | | | | | | | | |
| 0.62 | 0.93 | 0.056 | 1:1 | $C_{30}H_{24}N_5O_3SSiCl$ | 162 | 60.13 | 3.97 | 11.65 | 5.45 | 5.88 | 4.58 | 576 |
| (2.45) | (2.45) | (2.43) | | Orange solid | | (60.24) | (4.04) | (11.71) | (5.36) | (5.93) | (4.69) | (598.15) |
| $Ph_2SiCl_2$ | $L_4H$ | | | | | | | | | | | |
| 0.70 | 2.11 | 0.127 | 1:2 | $C_{48}H_{38}N_{10}O_6S_2Si$ | 285 | 61.02 | 4.15 | 14.76 | 6.67 | – | 2.83 | 928 |
| (2.76) | (5.53) | (5.52) | | Light orange solid | | (61.13) | (4.06) | (14.85) | (6.80) | | (2.98) | (943.10) |
| $Me_2SiCl_2$ | $L_4H$ | | | | | | | | | | | |
| 0.88 | 2.60 | 0.156 | 1:1 | $C_{20}H_{20}N_5O_2SSiCl$ | 118 | 50.56 | 4.12 | 14.63 | 6.65 | 7.40 | 5.84 | 455 |
| (6.82) | (6.82) | (6.79) | | Orange solid | | (50.68) | (4.25) | (14.77) | (6.76) | (7.48) | (5.93) | (474.01) |
| $Me_2SiCl_2$ | $L_4H$ | | | | | | | | | | | |
| 0.53 | 3.13 | 0.188 | 1:2 | $C_{38}H_{34}N_{10}O_6S_2Si$ | 98 | 55.66 | 4.04 | 17.21 | 7.75 | – | 3.33 | 796 |
| (4.11) | (8.21) | (8.17) | | Orange solid | | (55.73) | (4.18) | (17.10) | (7.83) | | (3.43) | (818.96) |

a = Calculated values are given in parenthesis

**Table II**

IR Spectral Data of the Ligands and their Corresponding Silicon Complexes.

| Compound | νN-H | νS-H | νC=N | ν Si ← N | ν Si-S/Si-O | ν Si - Cl | $νNO_2$ |
|---|---|---|---|---|---|---|---|
| $L_1H$ | 3100 | - | 1615 | - | - | - | - |
| $Me_2SiCl$ ($L_1$) | - | - | 1610 | 576 | 618 | 516 | - |
| $Me_2Si$ $(L_1)_2$ | - | - | 1602 | 580 | 620 | - | - |
| $L_2H$ | 3142 | 2525 | 1613 | - | - | - | - |
| $Me_2SiCl(L_2)$ | - | - | 1600 | 582 | 538 | 510 | - |
| $Me_2Si(L_2)_2$ | - | - | 1598 | 578 | 545 | - | - |
| $L_3H$ | 3250 | 2527 | 1612 | - | - | - | 1345 (sym)<br>1520 (asym) |
| $Ph_2SiCl$ ($L_3$) | - | - | 1602 | 580 | 543 | 512 | 1344 (sym)<br>1516 (asym) |
| $Ph_2Si(L_3)_2$- | - | - | 1595 | 579 | 540 | - | 1345 (sym)<br>1520 (asym) |
| $L_4H$ | 3186 | 2526 | 1614 | - | - | - | 1342 (sym)<br>1520 (asym) |
| $Ph_2SiCl$ ($L_4$) | - | - | 1601 | 583 | 542 | 515 | 1343 (sym)<br>1521 (asym) |
| $Ph_2Si$ $(L_4)_2$ | - | - | 1596 | 577 | 536 | - | 1342 (sym)<br>1518 (asym) |

and amido-oxygen/thiolosulphur lends further support to the proposed coordination in these complexes. The new carbon signals due to Si–Me/Si–Ph are also observed and reported. The carbon resonances of these complexes were assigned with the help of off resonance spectra and standard literature /22-24/.

### $^{29}Si$ NMR Spectra

The $^{29}Si$ NMR spectra (Table III) of 1 : 1 and 1 : 2 silicon complexes give sharp signals in the range of δ –92.05 – 95.02 ppm and δ –102.12 – 125.00 ppm which is an indicative of penta- and hexa-coordinated environments, respectively /25-27/, around the silicon atom.

Thus, on the basis of the foregoing spectral features and monomeric behaviour of the complexes, the following penta-coordinated trigonal bipyramidal and hexacoordinated octahedral geometries, have been suggested for the 1 : 1 and 1 : 2 (Fig. 2) derivatives, respectively.

**Table III**

$^1$H NMR Spectral Data (δ, ppm) of the Ligands and Their Corresponding Silicon Complexes

| Compound | HC-C=N (s) | -NH (ring) (bs) | -NH (free) (bs) | $-NH_2$ (bs) | Aromatic (m) | Si-Me/Ph | $^{29}$Si |
|---|---|---|---|---|---|---|---|
| $L_1H$ | 2.13 | 12.32 | 10.08 | 2.42 | 7.24 - 6.08 | - | |
| $Me_2SiCl$ $(L_1)$ | 2.22 | 12.38 | - | 2.36 | 7.68 - 6.65 | 0.98 | -102.12 |
| $Me_2Si(L_1)_2$ | 2.25 | 12.36 | - | 2.44 | 7.88 - 6.74 | 0.74 | -93.24 |
| $L_2H$ | 2.20 | 12.80 | 10.32 | 3.46 | 7.94 - 7.54 | - | - |
| $Me_2SiCl(L_2)$ | 2.27 | 12.76 | - | 3.35 | 7.92 - 6.76 | 0.72 | -92.05 |
| $Me_2Si(L_2)_2$ | 2.26 | 12.78 | - | 3.07 | 7.94 - 6.54 | 0.80 | -112.72 |
| $L_3H$ | 2.24 | 11.08 | 10.06 | 3.14 | 7.94 - 5.62 | - | - |
| $Ph_2SiCl(L_3)$ | 2.26 | 11.24 | - | 3.32 | 8.28 - 7.38 | * | -94.56 |
| $Ph_2Si(L_3)_2$ | 2.25 | 11.38 | - | 3.46 | 8.08 - 7.56 | * | -125.00 |
| $L_4H$ | 2.22 | 11.12 | 10.24 | 2.64 | 7.70 - 6.32 | - | - |
| $Ph_2SiCl(L_4)$ | 2.23 | 11.22 | - | 2.72 | 8.02 - 6.72 | * | -95.02 |
| $Ph_2Si(L_4)_2$ | 2.25 | 11.26 | - | 2.68 | 8.10 - 6.66 | * | -116.38 |

s = singlet; bs = broad singlet; m = multiplet; * = merged with aromatic protons

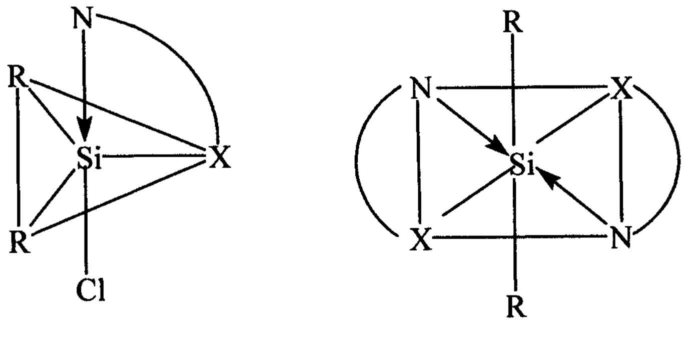

1:1 1:2

**Fig. 2**

Where, R = Me or Ph and N⌒X = donor site of the ligand molecule.

**Table IV**

$^{13}C$ NMR Spectral Data (δ, ppm) of the Ligands and Their Corresponding Silicon Complexes

| Compound | Amido/Thiolo | Azomethine | NH–C=O | Aromatic | Si–Me/Ph |
|---|---|---|---|---|---|
| $L_1H$ | 180.52 | 159.92 | 165.86 | 141.24, 126.16, 128.94, 129.94, 126.16, 124.08 | – |
| $Me_2SiCl (L_1)$ | 167.14 | 153.22 | 164.38 | 143.82, 127.96, 120.30, 129.56, 126.88, 125.18 | 14.66 |
| $Me_2Si (L_1)_2$ | 166.96 | 148.20 | 164.88 | 144.08, 128.88, 126.66, 123.46, 125.48, 125.66 | 15.45 |
| $L_2H$ | 169.65 | 158.92 | 165.86 | 141.29, 126.72, 129.10, 129.64, 123.52, 123.14 | – |
| $Ph_2SiCl (L_2)$ | 164.32 | 152.86 | 163.44 | 143.82, 127.96, 126.88, 123.32, 122.36, 120.66 | 138.18, 137.33, 134.26, 130.33 |
| $Ph_2Si(L_2)_2$ | 164.12 | 150.18 | 162.72 | 143.92, 127.86, 126.66, 123.46, 122.48, 120.66 | 137.24, 136.17, 137.86, 139.32 |
| $L_3H$ | 177.60 | 156.72 | 167.50 | 148.08, 144.05, 136.25, 135.71, 133.22, 132.13 | – |
| $Ph_2Si(L_3)_2$ | 169.98 | 154.82 | 167.22 | 145.46, 135.04, 129.45, 126.24, 127.52, 122.95 | 131.12, 134.55, 133.68, 136.74 |
| $L_4H$ | 171.54 | 155.10 | 167.58 | 147.24, 144.28, 136.92, 135.72, 130.22, 129.71 | – |
| $Me_2Si(L_4)_2$ | 167.36 | 151.88 | 165.51 | 146.75, 138.82, 132.54, 128.22, 125.94, 123.55 | 13.45 |

### Fungicidal and Bactericidal Activities

Fungicidal and bactericidal activities of the ligands and their respective diorganosilicon(IV) complexes against pathogenic fungi and bacteria are recorded in Table V. It is apparent that all the complexes showed better antimicrobial activity than their parent ligands and also, sulphur containing compounds are more toxic than the oxygen containing compounds. Among the various compounds, diphenylsilicon complexes of 1,3-dihydro-3-[2(4-nitrophenyl)-2-oxoethylidene]-2H-indol-2-one-hydrazinecarbothioamide ($L_3H$) demonstrated comparable inhibitory action than the conventional fungicide, Bavistin and better inhibitory action to the conventional bactericide, Streptomycin. The enhanced antimicrobial activity of the silicon chelates over their corresponding starting materials can be well explained from a purely scientific point of view. Here, we have distinguished different methods by which complexes can exert their action.

(1) According to Tweedy /28/, chelation reduces the polarity of the metal ion mainly because of partial sharing of its positive charge with the donor groups and possible π-electron delocalisation within the

**Table V**

Antimicrobial Data of the Ligands and Their Corresponding Silicon Complexes

| Compound | Antifungal Screening Data: Average percentage inhibition after 95 h (conc. in ppm) | | | | | | Antibacterial Screening Data: Diameter of inhibition zone (mm) after 24 h (conc. in ppm) | | | |
|---|---|---|---|---|---|---|---|---|---|---|
| | *Fusarium oxysporum* | | | *Aspergillus niger* | | | *Klebsiella aerogenous* (–) | | *Zymomonas mobilis* (+) | |
| | **50** | **100** | **200** | **50** | **100** | **200** | **500** | **1000** | **500** | **1000** |
| $L_1H$ | 68 | 71 | 77 | 72 | 80 | 82 | 3 | 4 | 7 | 8 |
| $Ph_2SiCl(L_1)$ | 68 | 72 | 74 | 74 | 80 | 82 | 5 | 8 | 11 | 10 |
| $Ph_2Si(L_1)_2$ | 74 | 80 | 85 | 76 | 84 | 87 | 7 | 10 | 13 | 12 |
| $Me_2SiCl(L_1)$ | 69 | 73 | 80 | 72 | 81 | 84 | 4 | 6 | 10 | 9 |
| $Me_2Si(L_1)_2$ | 70 | 75 | 81 | 73 | 82 | 85 | 6 | 9 | 12 | 11 |
| $L_2H$ | 72 | 78 | 83 | 76 | 85 | 88 | 7 | 9 | 9 | 11 |
| $Ph_2SiCl(L_2)$ | 75 | 80 | 86 | 79 | 88 | 90 | 7 | 8 | 12 | 13 |
| $Ph_2Si(L_2)_2$ | 77 | 81 | 89 | 80 | 89 | 92 | 9 | 12 | 13 | 14 |
| $Me_2SiCl(L_2)$ | 72 | 80 | 85 | 77 | 86 | 89 | 5 | 8 | 12 | 11 |
| $Me_2Si(L_2)_2$ | 74 | 79 | 86 | 78 | 87 | 90 | 8 | 11 | 12 | 11 |
| $L_3H$ | 77 | 83 | 89 | 85 | 91 | 95 | 8 | 12 | 11 | 12 |
| $Ph_2SiCl(L_3)$ | 81 | 85 | 91 | 85 | 93 | 96 | 10 | 13 | 12 | 16 |
| $Ph_2Si(L_3)_2$ | 81 | 87 | 94 | 88 | 95 | 98 | 12 | 14 | 16 | 19 |
| $Me_2SiCl(L_3)$ | 78 | 85 | 91 | 86 | 91 | 96 | 9 | 12 | 11 | 12 |
| $Me_2Si(L_3)_2$ | 79 | 85 | 93 | 87 | 94 | 97 | 10 | 12 | 14 | 15 |
| $L_4H$ | 75 | 81 | 87 | 84 | 89 | 93 | 6 | 10 | 9 | 10 |
| $Ph_2SiCl(L_4)$ | 75 | 82 | 88 | 86 | 91 | 92 | 6 | 10 | 11 | 12 |
| $Ph_2Si(L_4)_2$ | 77 | 83 | 89 | 87 | 92 | 95 | 11 | 13 | 15 | 17 |
| $Me_2SiCl(L_4)$ | 75 | 81 | 87 | 86 | 90 | 91 | 5 | 9 | 10 | 11 |
| $Me_2Si(L_4)_2$ | 76 | 82 | 88 | 86 | 92 | 93 | 10 | 12 | 14 | 16 |
| Bavistin | 91 | 100 | 100 | 89 | 98 | 100 | – | – | – | – |
| Streptomycin | – | – | – | – | – | – | 3 | 5 | 15 | 17 |

whole chelate ring. This chelation increases the lipophilic nature of the metal complex which subsequently favours its permeation through the lipid layer of the cell membrane of the microorganism and thereby, impairing normal cell process.

(2) The mechanism of the toxicity of the complexes may also be due to the inhibition of the energy production or ATP production /29/; for instance by inhibition of respiration or by uncoupling of oxidative phosphorylation.

(3) Enzymes which require free sulphydryl groups (–SH) for activity appear to be especially susceptible to inactivation by the complexes. Due to the greater lipid solubility, the complexes facilitate their diffusion through membrane to the site of action and ultimately killing them by combining with the (–SH) groups of the cell enzymen /30/.

(4) In antibacterial activity, the complexes were more toxic towards Gram (+) strains than Gram (–) strains. The reason is the difference in the structure of the cell walls. The walls of Gram (–) cells are more complex than those of Gram (+) cells (lipopolysaccharides form an outer lipid membrane and contribute to the antigenic properties of Gram (–) cells).

### Toxicological Effects on Male Albino Rats

The ligand ($L_3H$) and its corresponding diphenyl-silicon complex which showed good antimicrobial activity and the conventional fungicide, Bavistin when exposed to male albino rats for 60 days at a dose level of 30 mg/kg.b.wt./day produced the following effects:

(1) It is observed from Table VI that the rats exposed to Bavistin, showed highly significant ($P \leq 0.01$ and $P \leq 0.001$) increase in alanine aminotransferase, aspartate aminotransferase and alkaline phosphatase in comparison to the control rats as well as the rats exposed to $L_3H$ and its diphenylsilicon complex. This may be due to the necrosis of hepatocytes which causes increase in the permeability of the cell membranes, resulting in the release of transferases into the blood stream. A significant increase ($P \leq 0.05$ and $P \leq 0.01$) in the level of the cholesterol and a significant decrease ($P \leq 0.05$ and $P \leq 0.01$) in the level of the serum protein and albumin was also observed in the Bavistin treated rats which may be related to cirrhosis of the liver, nephrotic syndromes or neoplastic diseases /31-33/. The present study finds support from the work of other toxicologists. Also, there is a significant increase ($P \leq 0.05$ and $P \leq 0.001$) in the urea, creatinine and uric acid level of the Bavistin treated rats but the rats exposed to $L_3H$ showed highly

**Table VI**

Serum Analyses of Rats Exposed to Bavistin, $L_3H$ and $Ph_2Si(L_3)_2$ Complex

| Treatment | Urea | Creatinine | Uric acid | Cholesterol | Alanine amino-transferase | Aspartate amino-transferase | Total Protein | Albumin | Alkaline phosphatase |
|---|---|---|---|---|---|---|---|---|---|
| | mg/dl | | | | units/ml | | g/L | | KA units |
| Control | 32.0 | 0.86 | 5.81 | 94.68 | 132.20 | 76.18 | 55.45 | 40.00 | 68.14 |
| (vehicle treated) | ± 1.26 | ±0.15 | ±0.15 | ±6.34 | ±7.00 | ±2.31 | ±0.05 | ±2.64 | ±0.86 |
| Bavistin | 36.75* | 1.56** | 7.06* | 126.14* | 186.32*** | 119.26*** | 51.12* | 23.05* | 80.92* |
| | ±0.46 | ±0.12 | ±0.43 | ±5.68 | ±4.56 | ±3.82 | ±0.40 | ±0.40 | ±1.22 |
| $L_3H$ | 42.18* | 1.45** | 6.97** | 102.43* | 147.62* | 77.13 | 53.82 | 37.71 | 69.93 |
| | ±1.07 | ±0.13 | 0.33 | ±1.67 | ±1.55 | ±1.72 | ±0.17 | ±1.15 | ±0.52 |
| $Ph_2Si(L_3)_2$ | 41.13* | 1.18 | 6.66 | 100.24 | 136.14 | 74.66 | 54.16 | 36.91 | 69.13 |
| | ±0.68 | ±0.06 | ±0.17 | ±1.32 | ±1.47 | ±3.51 | ±0.06 | ±0.61 | ±0.17 |

**Table VII**

Blood Analyses of Rats Exposed to Bavistin, $L_3H$ and $Ph_2Si(L_3)_2$ Complex

| Treatment | Total Erythrocyte Count (TEC) | Total Leukocyte Count (TLC) | Hemoglobin | Hematocrit |
|---|---|---|---|---|
| | million/mm$^3$ | mm$^3$ | gm% | % |
| Control (vehicle treated) | 6.56 ± 0.21 | 5300 ± 253.34 | 15.25 ± 0.32 | 50.44 ± 1.59 |
| Bavistin | 5.04 ± 0.16** | 8215.00 ± 232.73*** | 11.14 ± 0.18** | 38.76 ± 0.52** |
| $L_3H$ | 5.90 ± 0.28 | 6926.38 ± 135.46* | 13.16 ± 0.45 | 41.57 ± 0.88* |
| $Ph_2Si(L_3)_2$ | 5.95 ± 0.44 | 5828.65 ± 168.22 | 13.95 ± 1.16 | 45.32 ± 2.06 |

significant ($P \leq 0.01$ and $P \leq 0.001$) increase in these parameters which is an indicator of the impaired renal function. It is also observed, that the increase in the urea, uric acid and creatinine is more pronounced in the rats exposed to the ligand than the silicon complex.

(2) Significant reduction in the total erythrocyte count ($P \leq 0.01$), hemoglobin ($P \leq 0.01$) and hematocrit percent ($P \leq 0.01$) values and significant increase ($P \leq 0.001$) in leukocyte count are more pronounced in rats exposed to Bavistin than $L_3H$ and its silicon complex (Table VII). It is also observed that the ligand $L_3H$ showed less decrease in the total erythrocyte count, hemoglobin and hematocrit percent value than its silicon complex. This may be due to the histopathological destruction of the liver and kidney so as to reduce the availability of the erythropoitin, which is produced in the juxtaglomerular apparatus in the kidney and is secreted in the plasma for the utilisation by the stem cells of the bone marrow.

(3) The motility of spermatozoa in cauda epididymis is decreased by 60.29% in the case of rats exposed to Bavistin, 27.98% in the case of $L_3H$ and 32.83% in the case of diphenylsilicon complex as compared to the control rats (Table VIII). This may be due to an alteration in the enzymatic activities of the oxidative phosphorolytic process required for ATP production which in turn is necessary for the forward movement of spermatozoa /34,35/. It is observed that the silicon complex caused more reduction in sperm motility and density in testes and cauda epididymis than the ligand $L_3H$. Reduction in the sperm counts in testes and cauda epididymis may be either due to the altered gonadotrophins (LH and FSH) necessary for normal sperm production, development and maturation /36,37/ or altered androgen metabolism.

Thus, it has been concluded that the silicon complex of the ligand, $L_3H$, showed less toxic effects on parameters related to serum biochemistry, hematology and fertility than the respective ligand $L_3H$ which in turn showed less toxic effects than Bavistin i.e. ligand $L_3H$ and its corresponding diphenylsilicon complex showed less toxicity related to the liver and kidney function in comparison to the rats exposed to Bavistin. It has also been revealed that Bavistin showed more antifertility effect than the complex which in turn showed more antifertility effect than the ligand.

**Table VIII**

Spermdynamics and Fertility of Rats Exposed to Bavistin, $L_3H$ and $Ph_2Si(L_3)_2$ Complex

| Treatment | Sperm Mobility (%) | Sperm Density (million/ml) | | Fertility (%) |
|---|---|---|---|---|
| | | Testes | Cauda epididymis | |
| Control (vehicle treated) | 69.61 ± 4.34 | 4.15 ± 1.86 | 21.70 ± 2.06 | 100 (+)ve |
| Bavistin | 27.64 ± 2.35 *** | 0.86 ± 0.06*** | 10.13 ± 1.72 * | 80 (−)ve |
| $L_3H$ | 50.13 ± 0.97 | 1.73 ± 0.12* | 14.52 ± 1.36 | 55 (−)ve |
| $Ph_2Si(L_3)_2$ | 46.76 ± 1.17* | 1.09 ± 0.07** | 10.19 ± 1.83* | 60 (−)ve |

z(Mean ± SEM of 5 animals)

* = $P \leq 0.05$

** = $P \leq 0.01$

*** = $P \leq 0.001$

### EXPERIMENTAL

Adequate care was taken to keep the organosilicon(IV) complexes, chemicals and glass apparatus free from moisture. Clean and well dried glass apparatus fitted with quickfit interchangeable standard ground joints was used throughout the experimental work. Chemicals and solvents used were dried and purified by standard methods.

### Preparation of the Imines

The ligands were prepared by the condensation of 1,3-dihydro-3-[2-(phenyl)-2-oxoethylidene]-2H-indol-2-one with hydrazinecarboxamide hydro-chloride (in the presence of an equimolar quantity of sodium acetate) and hydrazinecarbothioamide in 1:1 molar ratio in the ethanol. The other hydrazinecarbothioamides were prepared by the condensation of 1,3-dihydro-3[2-(4-nitrophenyl)2-oxoethylidene]-2H-indol-2-one and 1,3-dihydro-3-[2-(4-nitro-3-methylphenyl)-2-oxoethylidene]-2H-indol-2-one with hydrazinecarbo-thioamide in 1:1 molar ratio in ethanol. After refluxing, the contents were separated out as crystalline solids. These were dried and purified by the recrystallisation from the same solvent. The melting points (°C) of these imines are: $L_1H$, 180°C; $L_2H$, 176°C; $L_3H$, 185°C and $L_4H$, 162°C

### Synthesis of the Complexes

To a weighed amount of diorganosilicon(IV) dichlorides ($Ph_2SiCl_2$ and $Me_2SiCl_2$) in dry methanol was added the corresponding amount of the sodium salt of the ligands $L_1H$, $L_2H$, $L_3H$ and $L_4H$ in 1:1 and 1:2

molar ratios. The mixture was refluxed for 12-15 h on a fractionating column. The sodium chloride formed during the reaction was removed by filtration and the filtrate was dried under reduced pressure. The product was purified by repeated washing with (1:1) mixture of dry methanol and cyclohexane.

### Physical Measurements and Analytical Methods

Carbon and hydrogen analyses of the compounds as well as the parent ligands were performed at the RSIC Chennai and Central Drug Research Institute, Lucknow. Nitrogen, sulphur and chlorine were estimated by Kjeldahl's, Messenger's and Volhard's methods /38/, respectively. Silicon was determined gravimetrically as $SiO_2$. Molecular weights were determined by the Rast Camphor method /39/. The purity of the compounds was checked by TLC. The IR spectra were recorded as KBr pellets or Nujol mulls using a model Nicolet Magna FTIR-550 spectrophotometer. $^{1}H$, $^{13}C$ and $^{29}Si$ NMR spectra were scanned on Jeol FX90Q spectrometer in DMSO-$d_6$ for $^{1}H$ NMR and methanol for $^{13}C$ and $^{29}Si$ NMR, using tetramethyl silane as an internal standard.

### Biocidal Screening

Bioefficacies of the synthesized ligands and their corresponding organosilicon complexes were evaluated *in vitro* against a variety of fungi and bacteria and *in vivo* against male albino rats.

### *Fungicidal and Bactericidal Activities*

The *in vitro* growth inhibitory activity of the synthesized compounds was tested against pathogenic fungi, viz. *Fusarium oxysporum* and *Aspergillus niger* and pathogenic bacteria, viz. Gram negative, *Klebsiella aerogenous* and Gram positive, Zymomonas mobilis. Adequate temperature, requisite nutrient and growth media free from other microorganisms were employed for the growth of cultures of both fungi and bacteria /40/. The conventional fungicide, *Bavistin* and bactericide, *Streptomycin* were used as standards for comparing the activity of the compounds. The Radial Growth Method and Paper Disc Method were used to evaluate the antifungal and antibacterial activities, respectively /41/.

### *Toxicological Effects on Male Albino Rats*

The ligand and the complex showing good antimicrobial activity were chosen for oral administration to the male albino rats for 60 days. Bavistin was used as standard for the comparison. Twenty adult male albino rats of inbred colony (body weight 80-100 gm) divided into four groups of five animals each, were maintained in an air-conditioned animal house at 24°C ± 2°C with 14 hours light and fed with balanced pellet diet and water *ad libitum.*

The first group served with vehicle (olive oil) was treated as control. The animals of the second, third and fourth groups received 30 mg/kg.b.wt./day suspended in 0.2 ml olive oil of Bavistin, ligand ($L_3H$) and diphenylsilicon complex, respectively. The animals were weighed and autopsied on the 61$^{st}$ day under light ether anaesthesia and the blood from the heart was collected in pre-heparinized tubes for hematological studies. Serum was obtained from blood by centrifugation at 3000 rpm and stored at –20°C for biochemical estimation, done colorimetrically at a wavelength of 540 and 620 nm. Fertility test (spermdynamics) was also performed by using Neubaur's hemocytometer to check the potency of the compounds.

### ACKNOWLEDGEMENT

The authors are thankful to the University Grant Commission, Bahadur Shah Zafar Marg, New Delhi, India for financial assistance through Grant No. F.12-18/2004 (SR).

### REFERENCES

1. A. Saxena and J.P. Tandon, *Polyhedron.* **3**, 681 (1985).
2. T.N. Srivastava and M.A. Siddiqui, *J. Indian Chem., Soc.,* **64**, 500 (1987).
3. M.A. Ali, K.K. Dey, M. Nazimuddin, F.E. Smith, R.J. Butcher, J.P. Jasinski and J.M. Jasinski, *Polyhdedron,* **15**, 3331 (1996).
4. D.Kovala-Demertzi, J.R.Miller, N. Kourkoumelis, S.K. Hadjikakou, M.A.Demertzis, *Polyhedron,* **18**, 1005 (1999).
5. D.X. West, S.B. Padhye and P.B. Sonawane, *Structure Bonding,* **76**, 1 (1991) and refs. therein.
6. D.X. West and N.M.Kozub, *Trans. Met. Chem.,* **21**, 52 (1996) and refs. therein.
7. J. Aasen and G. Haukeness, *Acta Pathol. Microbiol. Scand. Sect. B,* **80**, 246 (1972); *Chem. Abstr.,* **77**, 57063 (1972).
8. M. Tonew, E. Tonew and L. Heinish, *Acta Virol.,* **18**, 17 (1974).
9. I. Susheela, B. Singh, H.M. Dani, M.K.P. Amma and K. Sareen, *Enzymologia,* **37**, 325 (1969); *Chem. Abstr.* **72**, 74963 (1970).
10. H.R. Chen, A.W. Galston and L. Milstone, *Plant Physiol.,* **41**, 1485 (1966); S. Mukherji and T. Roy, *Indian J. Exp. Biol.* **10**, 395 (1972).
11. A. Scozzafava and C.T. Supuran, *J. Med. Chem.,* **43**, 3677 (2000).
12. C.T. Supuran, A. Scozzafava, L. Menabuoni, F. Minicione, F. Briganti and G. Mincione, *Metal Based Drugs,* **6**, 67 (1999).
13. C.T. Supuran, A. Scozzafava, I. Saramet, M.D. Banciu, *J. Enzyme Inhib.,* **13**, 177 (1998).
14. C.T. Supuran, F. Mincione, A. Scozzafava, F. Briganti, G. Mincione and M.A. Ilies, *Eur. J. Med. Chem.,* **33**, 247 (1998).
15. C. Eaborn, K. Izod and J.D. Smith, *J. Organomet. Chem.,* **500**, 89 (1995).
16. H.B. Singh and J.P. Tandon, *Synth. React. Inorg. Met.-Org. Chem.,* **15**, 391 (1985).
17. K.M. Kadish, Q.Y.Xu, J.M. Barbe and R. Guilard, *Inorg. Chem.,* **27**, 1191 (1988).
18. C. Gliowell and D.W.H. Rankin, *J. Chem. Soc.,* A, **1969**, 753.
19. E.A.V. Ebsworth and M.J. Mays, *J. Chem. Soc.,* **1964**, 3450.

20. A.L. Smith, *Spectrochim. Acta,* **16**, 87 (1960).
21. P.S. Kalsi, *Spectroscopy of Organic Compounds,* 5[th] Ed., New Age International Publ. Ltd., New Delhi, 2002.
22. Savita Belwal and R.V. Singh, *Appl. Organomet. Chem.*, **12**, 39 (1998).
23. Savita Belwal R.K. Saini and R.V. Singh, *Indian J. Chem.*, **37A**, 245 (1998).
24. E. Pretsch, T. Clerc, J. Seibi and W. Simon, *Tables of Organic Compounds,* 2[nd] Ed., Springer Verlag, P.C., 1989; p. 120.
25. R.R. Gupta and R. Kaushal, *J. Indian Chem. Soc.*, **LIII**, 552 (1976).
26. M.A. Ali and S.E. Livingstone, *Coord. Chem. Rev.*, **13**, 101 (1974).
27. J.D. Cargioli and E.A. Williams, *J. Organomet.Chem.*, **244**, 5 ( 1983).
28. B.G. Tweedy, *Phytopathology,* **55**, 910 (1964).
29. R.W. Marsh, *Systemic Fungicides,* Longman, Group Limited, 1972; p.150.
30. Nadira Wasi and H.B. Singh, *Inorg. Chim. Acta,* **151**, 287 (1988).
31. R.C. Gupta, *Ph.D. Thesis, Punjab Agriculture University,* Ludhiana, India (1977).
32. I. Chakravarty and R. Sreedhar, *IRCS, Med. Sci.*, **9**, 1115 (1981).
33. R.A. Young and H.M. Mehendale, *Fund. Chem. Toxicol.*, **24**, 863 (1986).
34. J.M. Bedford, *Biol. Report.*, **28**, 108 (1983).
35. W.W. Iso and C.S. Lee, *Arch. Androl.*, **7**, 85 (1981).
36. H.W.G. Baker, R.J. Santen, H.G. Burges, D.M. Dekretsec, B. Hudsen and R.J.Pepperell, *J. Steroid. Bio. Chem.*, **6**, 793 (1975).
37. M.C. Bastias, H.Kamijo and S.N. Povlou, *Fertil. Steril.* **59**(6), 1261 (1993).
38. A.I. Vogel, A *Textbook of Quantitative Chemical Analysis,* 5[th] Edn., Longman, ELBS London, 1989.
39. A.I. Vogel, *A Textbook of Practical Organic Chemistry,* 4[th] Ed., Longman, ELBS London, 1978; p. 323.
40. M. Dudeja, R. Malhotra and K.S. Dhindsa, *Synth. React. Inorg. Met.-Org. Chem.*, **23**, 921 (1993).
41. P. Nagpal, A. Gajraj, S.C. Joshi and R.V. Singh, *Appl. Organomet. Chem.*, **16**, 713 (2002).

